# A recurrent clonally distinct Burkitt lymphoma case highlights genetic key events contributing to oncogenesis

**DOI:** 10.1002/gcc.22743

**Published:** 2019-03-27

**Authors:** Dominique Penther, Pierre‐Julien Viailly, Sylvain Latour, Pascaline Etancelin, Elodie Bohers, Hélène Vellemans, Vincent Camus, Anne Lise Menard, Sophie Coutant, Hélène Lanic, Emilie Lemasle, Fanny Drieux, Liana Veresezan, Philippe Ruminy, Anna Raimbault, Jean Soulier, Thierry Frebourg, Hervé Tilly, Fabrice Jardin

**Affiliations:** ^1^ Department of Biopathology Centre Henri Becquerel Rouen France; ^2^ INSERM U1245 Centre Henri Becquerel and Rouen University Rouen France; ^3^ INSERM UMR_S1163 Institut Imagine Université Paris Descartes Paris France; ^4^ Department of Clinical Hematology Centre Henri Becquerel Rouen France; ^5^ Department of Genetics Rouen University Hospital, F76000 and Normandie Univ, UNIROUEN, Inserm U1245, Normandy Centre for Genomic and Personalized Medicine Rouen France; ^6^ INSERM U944/CNRS UMR7212 Saint Louis Hospital Paris France

**Keywords:** Burkitt lymphoma, EBV, *FANCM*, *MYC*, somatic mutation, whole exome sequencing

## Abstract

Burkitt lymphoma (BL) is characterized by a translocation of the *MYC* oncogene that leads to the upregulation of *MYC* expression, cell growth and proliferation. It is well‐established that *MYC* translocation is not a sufficient genetic event to cause BL. Next‐generation sequencing has recently provided a comprehensive analysis of the landscape of additional genetic events that contribute to BL lymphomagenesis. Refractory BL or relapsing BL are almost always incurable as a result of the selection of a highly chemoresistant clonally related cell population. Conversely, a few BL recurrence cases arising from clonally distinct tumors have been reported and were associated with a favorable outcome similar to that reported for first‐line treatment. Here, we used an unusual case of recurrent but clonally distinct EBV+ BL to highlight the key genetic events that drive BL lymphomagenesis. By whole exome sequencing, we established that *ID3* gene was targeted by distinct mutations in the two clonally unrelated diseases, highlighting the crucial role of this gene during lymphomagenesis. We also detected a heterozygous E1021K *PIK3CD* mutation, thus increasing the spectrum of somatic mutations altering the PI3K signaling pathway in BL. Interestingly, this mutation is known to be associated with activated phosphoinositide 3‐kinase delta syndrome (APDS). Finally, we also identified an inherited heterozygous truncating c.5791CT *FANCM* mutation that may contribute to the unusual recurrence of BL.

## 1 INTRODUCTION

Burkitt lymphoma (BL) is characterized by a translocation of the *MYC* oncogene that leads to the upregulation of *MYC* expression, cell growth and proliferation. It is well‐established that *MYC* translocation is not a sufficient genetic event to cause BL.^1^, ^2^, ^3^, ^4^ Next‐generation sequencing (NGS) has recently provided a comprehensive analysis of the landscape of additional genetic events that contribute to BL lymphomagenesis. Additional recurrent mutations are observed in the genes encoding TCF3 and its negative regulator, ID3, with up to 70% of tumors bearing mutations in one or both of the genes, suggesting that TCF3/ID3 play a key role in BL lymphomagenesis.^2^, ^4^ Furthermore, Epstein‐Barr virus (EBV), a ubiquitous oncogenic virus, is associated with B‐cell lymphomas, including BL and Hodgkin lymphoma.^5^ The role of EBV in BL is still unclear, and it has been suggested that EBV‐positive (EBV+) and ‐negative cases might arise from different cells of origin. EBV+ BL may arise from late germinal center lymphoblast memory B‐cells and EBV− BL from an earlier stage of differentiation. Despite its aggressiveness, BL treated in first‐line by intensive combination chemotherapy with rituximab and central nervous system (CNS) preventative procedures has resulted in event‐free survival rates of 80% to 90%.^6^ By contrast, refractory BL or relapsing (R/R) BL are almost always incurable as a result of the selection of highly chemoresistant clonally related cell populations. Conversely, a few BL recurrences arising from clonally distinct tumors have been reported and have been associated with a favorable outcome similar to that reported of first line treatment.^7^, ^8^, ^9^, ^10^, ^11^ Here, we used an unusual case of recurrent but clonally distinct EBV+ BL to highlight the key genetic events that drive BL lymphomagenesis.

## 2 CASE REPORT

An HIV‐negative Caucasian male individual initially presented in January 2015 at the age of 25 years with adenopathy and vena cava superior syndrome. No history of recurrent pulmonary infection/herpes virus infections or chronic inflammatory disease was reported. After lymph node biopsy, a diagnosis of BL with a typical immunophenotype was confirmed (BL1). Conventional cytogenetics showed that the tumor harbored the characteristic translocation t(8;22)(q24;q11) that juxtaposes the *MYC* and *IGL* loci with the loss of Y as an additional cytogenetic alteration (Supporting Information Figure S1A). Staging indicated stage IV disease without any involvement of the bone marrow or CNS (Supporting Information Figure S2A). The patient was treated with dose‐dense chemotherapy and rituximab, followed by autologous stem cell transplantation (ASCT) conditioned by BEAM (carmustine, etoposide, cytarabine, and melphalan)^12^. A complete remission (CR) was obtained. Two years later, the patient presented with facial paralysis and abdominal pain. PET scan showed multiple enlarged lymph nodes and a massive infiltration of the kidneys (Supporting Information Figure S2B). Bone marrow biopsy displayed massive infiltration (around 30%) by typical BL cells, and the CNS was considered infiltrated. The diagnosis of Burkitt leukemia was confirmed (BL2). In this case, cytogenetics showed that the tumor harbored the characteristic translocation t(8;14)(q24;q32) that juxtaposes the *MYC* and *IGH* loci, with a short interstitial deletion of the long arm of chromosome 13 as an additional cytogenetic aberration (Supporting Information Figure S1B). The main immunological features of BL1 and BL2, both EBV+/BCL2 neg/BCL6+, are summarized in Supporting Information Table S1 and representative histopathologic pictures provided in Supporting Information Figure S3A/3B. The patient received two cycles of R‐HyperCVAD (rituximab, cyclophosphamide, dexamethasone, methotrexate, doxorubicin, vincristine, cytarabine) with intrathecal methotrexate injection. CR was obtained, and the patient underwent an allogeneic transplantation from an HLA‐identical sibling donor that was conditioned by a myeloablative regimen (cyclophosphamide and total‐body irradiation). To date, the patient is considered in CR, with a follow‐up of 18 months. No abnormal toxicities, including unusual prolonged pancytopenia or mucosal toxicity, were observed throughout the different therapeutic sequences.

## 3 MATERIAL AND METHODS

### 3.1 Routine procedures

Conventional cytogenetics, EBV expression analysis and immunohistochemistry were performed using routine procedures. CDR3 sequence and VDJ analyses were performed using routine BIOMED2 procedures.

### 3.2 Whole exome sequencing and targeted sequencing

WES was performed on the DNA of BL1 and BL2 tumor tissues and compared to germline DNA from PBMCs obtained at the time of initial diagnosis. Data analysis was conducted as fully described in a previous work.^13^ Somatic variant calling was performed by VarScan, using germline DNA as a reference. Variant annotation was performed by GenerateReports software, as previously described.^14^ To confirm some genetic variants, BL1 and BL2 tumor DNA was also analyzed using our in‐house dedicated lymphopanel, as previously reported.^15^

Copy number analysis was done using the copynumber Bioconductor package [Nilsen G, Liestol K, Lingjaerde OC (2013); copynumber: Segmentation of single‐ and multi‐track copy number data by penalized least squares regression, R package version 1.20.0].

The *FANCM* and *PI3KCD* gene mutations reported in the index case report were identified using primers in a cohort of 29 additional BL or high‐grade B‐cell lymphoma (HGBCL) cases harboring a *MYC* translocation detected either by conventional cytogenetics and/or FISH. The primer sequences are provided in Supporting Information.

To investigate functionally the Fanconi anemia (FA) pathway integrity, hypersensitivity to mitomycin C (MMC) and the profile of FancD2 monoubiquitination were analyzed in primary fibroblast cells obtained from skin biopsy.^16^ The patient provided his consent for germline DNA sequencing.

## 4 RESULTS AND DISCUSSION

### 4.1 BL1 and BL2 are two clonally distinct diseases

To date, this report represents the most accurately described case of recurrent clonally distinct BL (see Supporting Information Table S2 for a summary of the previously published cases). This report provides the opportunity to highlight key molecular events that may contribute to BL oncogenesis. Conventional cytogenetics, VDJ/CDR3 sequence analysis and WES demonstrated without any ambiguity that BL1 and BL2 are two clonally distinct diseases that are sustained by distinct genetic events.

### 4.2 WES and targeted sequencing analysis demonstrated common and divergent alterations that drive BL oncogenesis

In addition to *MYC* translocation, WES studies have identified genetic mutations implicating pathways involved in BL oncogenesis. These alterations involved cell cycle and proliferation (*ID3*, *MYC*, *TP53*, *RET*, *DDX3X*, *TCF3*, *GAB1*, *CCND3*, etc.), nucleosome remodeling (*ARID1A* and *SMARCA4*), focal adhesion (*GNA13*, *RHOA*, and *ROCK1*) or PI3K signaling (*PI3KR1*, *EIF4B*, and *FGFR2*).^3^ We identified 64 acquired somatic variants in BL1 and 26 acquired variants in BL2. BL1 and BL2 harbored several mutations previously described that target the *MYC, TP53*, *SMARCA4, RHOA* and *ID3* genes (see Table 1 and Supporting Information). Of note, only *MYC* and *ID3* were mutated by distinct mutations in both BL1 and BL2. In BL1, the T73A *MYC* variant targets exon 2, whereas in BL2, the T8C variant was detected in exon 1. It is hypothesized that *MYC* mutations are a consequence of *MYC* translocation and somatic mutation processes under the control of Ig elements and may contribute to mRNA stability or MYC protein function. Because *ID3* mutations were observed in both BL1 and BL2, our observation reinforces the fact that these mutations represent a crucial step during BL oncogenesis. The BL2 variant is classified as a variant of uncertain significance. However, as previously reported, both variants occur in the highly conserved helix‐loop‐helix (HLH) functional domain of the protein, which is critical to its interactions with other HLH proteins.

**Table 1:** List of acquired somatic mutations detected by whole exome sequencing in the Burkitt lymphoma (BL) cases. Genes are classified by alphabetic order

Tumor	CHROM	Type	Gene	Exonic type	GenerateReports Class	SIFT score	Tumor variant frequency
BL1	chr9	Exonic	ABCA1	Stopgain	Uncertain significance	–	48.96%
BL1	chr11	Exonic	ADAMTS15	Nonsynonymous SNV	Likely pathogenic	0.001	33.33%
BL1	chr19	Exonic	ADGRL1	Nonsynonymous SNV	Uncertain significance	0.177	42.13%
BL1	chr17	Exonic	ALOX12B	Nonsynonymous SNV	Uncertain significance	1.0	35.56%
BL1	chr17	Exonic	AOC3	Nonsynonymous SNV	Likely pathogenic	0.001	47.96%
BL1	chr2	Exonic	BMP10	Nonsynonymous SNV	Likely pathogenic	0.0	50.3%
BL1	chr20	Exonic	BTBD3	Nonsynonymous SNV	Likely pathogenic	0.003	45.22%
BL1	chr4	Exonic	CASP6	Nonsynonymous SNV	Likely pathogenic	0.0	38.1%
BL1	chr18	Exonic	CDH19	Nonsynonymous SNV	Likely pathogenic	0.001	40%
BL1	chr9	Exonic	CDK5RAP2	Synonymous SNV	Uncertain significance		27.27%
BL1	chr1	Exonic	CLCA4	Nonsynonymous SNV	Uncertain significance	0.267	37.5%
BL1	chr2	Exonic	CNOT11	Nonsynonymous SNV	Likely pathogenic	0.002	44.05%
BL1	chr13	Exonic	COL4A1	Nonsynonymous SNV	Uncertain significance	0.313	41.75%
BL1	chr11	Exonic	DNHD1	Nonsynonymous SNV	Uncertain significance	0.729	20.2%
BL1	chr9	Exonic	FAM157B	Nonsynonymous SNV	Uncertain significance		16.67%
BL1	chr2	Exonic	FARSB	Nonsynonymous SNV	Likely pathogenic	0.0	41.1%
BL1	chr13	Exonic	FOXO1	Nonsynonymous SNV	Likely pathogenic	0.0	42.19%
BL1	chr5	Exonic	FYB	Nonsynonymous SNV	Uncertain significance	0.804	61.29%
BL1	chr11	Exonic	GAB2	Nonsynonymous SNV	Uncertain significance	0.153	45.79%
BL1	chr6	Exonic	GFRAL	Nonsynonymous SNV	Uncertain significance	0.0	45.92%
BL1	chr3	Exonic	GNAI2	Nonsynonymous SNV	Likely pathogenic	0.005	38.59%
BL1	chr15	Exonic	GOLGA6L3	Nonsynonymous SNV	Uncertain significance		23.08%
BL1	chr3	Exonic	GRIP2	Unknown	Likely pathogenic	–	37.78%
BL1	chr1	Exonic	HMCN1	Nonsynonymous SNV	Uncertain significance	0.592	38.98%
BL1	chr1	Exonic	HMCN1	Nonsynonymous SNV	Likely pathogenic	0.026	43.44%
BL1	chr1	Exonic	ID3	Nonsynonymous SNV	Likely pathogenic	0.0	39.59%
BL1	chr19	Exonic	LMTK3	Nonsynonymous SNV	Likely pathogenic	0.0	20%
BL1	chr19	Exonic	MED29	Nonsynonymous SNV	Uncertain significance	0.094	42.03%
BL1	chr2	Exonic	MEMO1	Nonsynonymous SNV	Uncertain significance	0.307	23.08%
BL1	chr2	Exonic	MEMO1	Nonsynonymous SNV	Uncertain significance	0.076	37.5%
BL1	chr8	Exonic	MYC	Nonsynonymous SNV	Likely pathogenic	0.037	41.95%
BL1	chr1	Exonic	NBPF10	Nonsynonymous SNV	Uncertain significance	–	18.75%
BL1	chr1	Exonic	NPR1	Nonsynonymous SNV	Likely pathogenic	0.001	38.89%
BL1	chr22	Exonic	PHF21B	Nonsynonymous SNV	Uncertain significance	0.033	44.62%
BL1	chr1	Exonic	PIK3CD	Nonsynonymous SNV	Likely pathogenic	0.002	48%
BL1	chr8	Exonic	PPP2R2A	Nonsynonymous SNV	Likely pathogenic	0.006	41.14%
BL1	chr4	Exonic	PPP2R2C	Nonsynonymous SNV	Uncertain significance	0.18	43.63%
BL1	chr1	Exonic	PRAMEF22	Nonsynonymous SNV	Uncertain significance		27.27%
BL1	chr4	Splicing	PRIMPOL	NA	Likely pathogenic	–	16.67%
BL1	chr7	Exonic	PTPRZ1	Nonsynonymous SNV	Likely pathogenic	0.029	41.46%
BL1	chr1	Exonic	RAP1GAP	Nonsynonymous SNV	Uncertain significance	0.142	38.41%
BL1	chr3	Exonic	RHOA	Nonsynonymous SNV	Uncertain significance	0.118	35.64%
BL1	chr3	Exonic	RHOA	Nonsynonymous SNV	Likely pathogenic	0.002	33.91%
BL1	chr5	Splicing	RNF145	NA	Likely pathogenic	–	50%
BL1	chr7	Exonic	RSPH10B	Nonsynonymous SNV	Likely pathogenic	0.031	18.18%
BL1	chr7	Exonic	SEMA3C	Nonsynonymous SNV	Likely pathogenic	0.004	35.71%
BL1	chr15	Exonic	SIN3A	Nonsynonymous SNV	Likely pathogenic	0.0	44.81%
BL1	chr15	Exonic	SIN3A	Nonsynonymous SNV	Likely pathogenic	0.0	44.26%
BL1	chr19	Exonic	SMARCA4	Nonsynonymous SNV	Likely pathogenic	0.0	49.11%
BL1	chr13	Exonic	TBC1D4	Stopgain	Likely pathogenic	–	51.29%
BL1	chr13	Exonic	TBC1D4	Stopgain	Uncertain significance	–	52.42%
BL1	chrX	Exonic	TENM1	Nonsynonymous SNV	Likely pathogenic	0.0	85.9%
BL1	chr15	Exonic	TLN2	Nonsynonymous SNV	Uncertain significance	0.291	42.96%
BL1	chr17	Exonic	TP53	Nonsynonymous SNV	Likely pathogenic	0.0	47.2%
BL1	chr18	Exonic	TRAPPC8	Nonsynonymous SNV	Uncertain significance	0.059	43.85%
BL1	chr13	Exonic	TRPC4	Nonsynonymous SNV	Uncertain significance	0.382	47%
BL1	chr15	Splicing	TRPM7	NA	Likely pathogenic	–	44.26%
BL1	chr11	Exonic	TUT1	Nonsynonymous SNV	Uncertain significance	1.0	48.63%
BL1	chr1	Exonic	USH2A	Nonsynonymous SNV	Uncertain significance	0.259	43.62%
BL1	chr15	Exonic	WDR72	Nonsynonymous SNV	Uncertain significance	0.091	39.73%
BL1	chr2	Exonic	XIRP2	Nonsynonymous SNV	Uncertain significance	0.014	42.31%
BL1	chr19	Exonic	ZNF555	Stopgain	Uncertain significance	–	47.37%
BL1	chr2	Exonic	ZSWIM2	Nonsynonymous SNV	Likely pathogenic	0.018	49.09%
BL1	chr6	Exonic	MOXD1	Nonsynonymous SNV	Likely pathogenic	0.002	40.54%
BL2	chr1	Exonic	IGFN1	Nonsynonymous SNV	Likely pathogenic	0.009	18.39%
BL2	chr6	Exonic	ADGB	Nonsynonymous SNV	Likely pathogenic	0.027	20%
BL2	chr5	Exonic	ANKRD31	Nonsynonymous SNV	Likely pathogenic	0.003	18.18%
BL2	chr12	Exonic	ANO6	Nonsynonymous SNV	Uncertain significance	1.0	16.67%
BL2	chr10	Exonic	ARMC4	Nonsynonymous SNV	Uncertain significance	0.1	25%
BL2	chr11	Exonic	C11orf74	Nonsynonymous SNV	Uncertain significance	0.031	16.95%
BL2	chr22	Exonic	CDC42EP1	Nonsynonymous SNV	Uncertain significance	0.006	18.18%
BL2	chr19	Exonic	CIC	Nonsynonymous SNV	Likely pathogenic	–	16.67%
BL2	chrX	Exonic	CYBB	Nonsynonymous SNV	Uncertain significance	0.167	18.18%
BL2	chr1	Exonic	DNAH14	Nonsynonymous SNV	Likely pathogenic	0.0	20%
BL2	chr9	Exonic	FAM102A	Nonsynonymous SNV	Uncertain significance	0.089	31.63%
BL2	chr1	Exonic	ID3	Stopgain	Uncertain significance	–	39.6%
BL2	chr22	Exonic	IGLL5	Nonsynonymous SNV	Uncertain significance		31.25%
BL2	chr1	Exonic	KIAA0754	Nonsynonymous SNV	Uncertain significance	–	16.67%
BL2	chr11	Exonic	KRTAP5‐4	Nonsynonymous SNV	Uncertain significance	0.03	25.81%
BL2	chr11	Exonic	MCAM	Nonsynonymous SNV	Uncertain significance	0.181	21.43%
BL2	chr3	Exonic	MUC20	Nonsynonymous SNV	Uncertain significance	0.0	16.67%
BL2	chr8	Exonic	MYC	Nonsynonymous SNV	Likely pathogenic	0.001	20%
BL2	chr14	Exonic	OR4E1	Nonsynonymous SNV	Uncertain significance		20%
BL2	chr7	Exonic	PLXNA4	Stopgain	Likely pathogenic	–	19.67%
BL2	chrX	Exonic	RBMX2	Nonsynonymous SNV	Uncertain significance	0.327	22.22%
BL2	chr8	Exonic	SPATC1	Nonsynonymous SNV	Uncertain significance	0.006	19.84%
BL2	chr6	Exonic	TRDN	Nonsynonymous SNV	Uncertain significance	0.007	18.18%
BL2	chr6	Exonic	TRDN	Nonsynonymous SNV	Uncertain significance	0.268	27.27%
BL2	chr7	Exonic	VGF	Nonsynonymous SNV	Uncertain significance	0.865	17.65%
BL2	chr4	Exonic	ZFP42	Nonsynonymous SNV	Uncertain significance	0.599	16.04%

Mutations are thought to alter the tumor suppressor ability of ID3, which is also a direct transcriptional target of *MYC*. Targeted sequencing using our in‐house lymphopanel confirmed *ID3* mutations with similar variant allele frequencies (44.26% in BL1 [p.I69S] and 46.7% in BL2 [p.Q71X]). Several mutations targeting genes involved in the PI3K signaling pathway, including *PIK3R1*, *GAB1*, *FGFR2*, and *EIF4B*, have been previously reported and suggest a cooperative role between MYC and the deregulation of PI3K signaling.^3^

To the best of our knowledge, we report here for the first time a heterozygous acquired mutation E1021K in *PIK3CD*, detected in BL1 (Table 1), thus increasing the spectrum of somatic mutations altering the PI3K signaling pathway in BL. Interestingly, this gain‐of‐function (GOF) mutation is known to be associated with activated phosphoinositide 3‐kinase delta syndrome (APDS).^17^ This inherited disorder, resulting from GOF mutations in *PIK3CD*, the gene encoding the p110δ catalytic subunit of phosphoinositide 3‐kinase (PI3KCδ), includes recurrent pulmonary infections (98%), nonneoplastic lymphoproliferations (75%), herpesvirus infections (49%) and autoinflammatory diseases (34%).^17^ Of note, four heterozygous GOF *PIK3CD* mutations (E1021K, N334K, E525K, and C416R) have been described, with E1021K being the most common. In the largest APDS cohort reported by Coulter and colleagues^17^, seven (13%) patients developed lymphoma between the ages of 18 months and 27 years. The cases were of various B‐cell histology, and two cases were considered EBV‐positive. To date, no BL case has been reported. Of note, in a cohort of 29 BL/HGBCL cases, we failed to detect any mutations targeting the PI3KCD kinase domain (Supporting Information). In addition, we identified several mutations that may contribute to lymphomagenesis or aggressiveness of the disease, including *SIN3A* (Sin3a causes the deacetylation of the MYC protein to directly repress MYC activity), *FOXO1*, *FYB*, and *GNAI2* (Table 1)^3^. Copy number variations (CNVs) were also analyzed and compared between BL1 and BL2. The list of CNVs is available in Supporting Information. In BL1, 133 CNV (53 losses, 80 gains) were identified (Supporting Information Table S5). CNV were mainly related to Immunoglobulin loci rearrangements and chromosome Y loss. No CNV was associated with somatic mutation of the second allele. Only seven CNV were retained in BL2, targeting genes of uncertain relevance in this setting. No CNV was shared by BL1 and BL2. This result suggests that *MYC* rearrangements and some mutations (*ID3*) are the key‐genetic events of the disease. Discrepancies between CNV detected by WES and conventional cytogenetics can be mostly explained by the sensibility of the two approaches. The Del (13)(q13q14) detected by conventional cytogenetics in BL2 and confirmed by FISH (data not shown) was not detected by the algorithm used for WES CNV analysis, reflecting most likely a percentage of tumor cells under the threshold of CNV detection sensibility.

### 4.3 Clinical and potential therapeutic relevance

Our case report confirms the crucial role of the PI3K/Akt/mTOR pathway in BL and the spectrum of mutations that contributes to its deregulation. Importantly, the PI3KCD inhibitor idelalisib has been investigated in a panel of BL cell lines, including cell lines that exhibit a high degree of resistance to both chemotherapy and anti‐CD20 immunotherapy, and demonstrate preclinical activity.^18^ Because PI3K/Akt is activated in EBV‐associated lymphoma by inducing BCR signaling, the mutation reported in an EBV+ BL suggests a synergistic effect able to alter this pathway. An oral dual inhibitor of PI3Kγ and PIK3CD (PI3Kδ), duvelisib, induces both apoptosis and cell cycle arrest in EBV‐positive and ‐negative B cell lines and reduces the expression of EBV lytic genes (BZLF1 and gp350/220) in EBV‐positive B cell lines.^19^ Our current observation therefore highlights new therapeutic opportunities in BL. Furthermore, a recently developed transgenic mouse model shows that the concurrent activation of both Myc and PI3K leads to lymphoid tumors that morphologically and genetically appear BL‐like, suggesting that the coordination of overexpression of *Myc* and activation of PI3K may contribute to the development of BL and represent key synergistic events during lymphomagenesis.^20^

### 4.4 Constitutional genetic background that may contribute to BL emergence

Because BL1 and BL2 were both EBV+ and EBV infection is considered as a risk factor for developing BL, we tried to detect some alterations in target genes that are involved in EBV immune response and favor EBV‐associated lymphoproliferative disorders (LPDs). Among these alterations are *SH2D1A (SAP), XIAP, ITK, MAGT1, CD27, CD70, CTPS1, RASGRP1,* and *CORO1A* deficiencies.^21^ None of these genes were found to be altered in BL1, BL2 or germline DNA, suggesting that an EBV immune response deficiency involving these genes cannot be considered responsible for this unusual phenotype.

We then sought more specifically to identify alterations in genes involved in DNA repair. We identified a heterozygous stop‐gain mutation (c.5791C>T; p.Arg1931*) in the *FANCM* gene. This mutation was also detected in the patient's sister, demonstrating that the mutation is inherited (see pedigree in Supporting Information Figure S4). At the resolution level of a WES approach we did not detect a *FANCM copy loss*. *FANCM* was identified in 2005 as a member of the FA core complex. Its product FANCM plays an important role in the FA pathway involved in DNA damage responses and repair.^22^ Interestingly, it has been demonstrated that the *FANCM* c.5791C>T nonsense mutation induces exon skipping, affects DNA repair activity and is a familial breast cancer risk factor.^23^, ^24^ The mutation causes an out‐of‐frame deletion of exon 22 due to the creation of a binding site for the pre‐mRNA processing protein hnRNP A1.^24^

**Figure 1:**
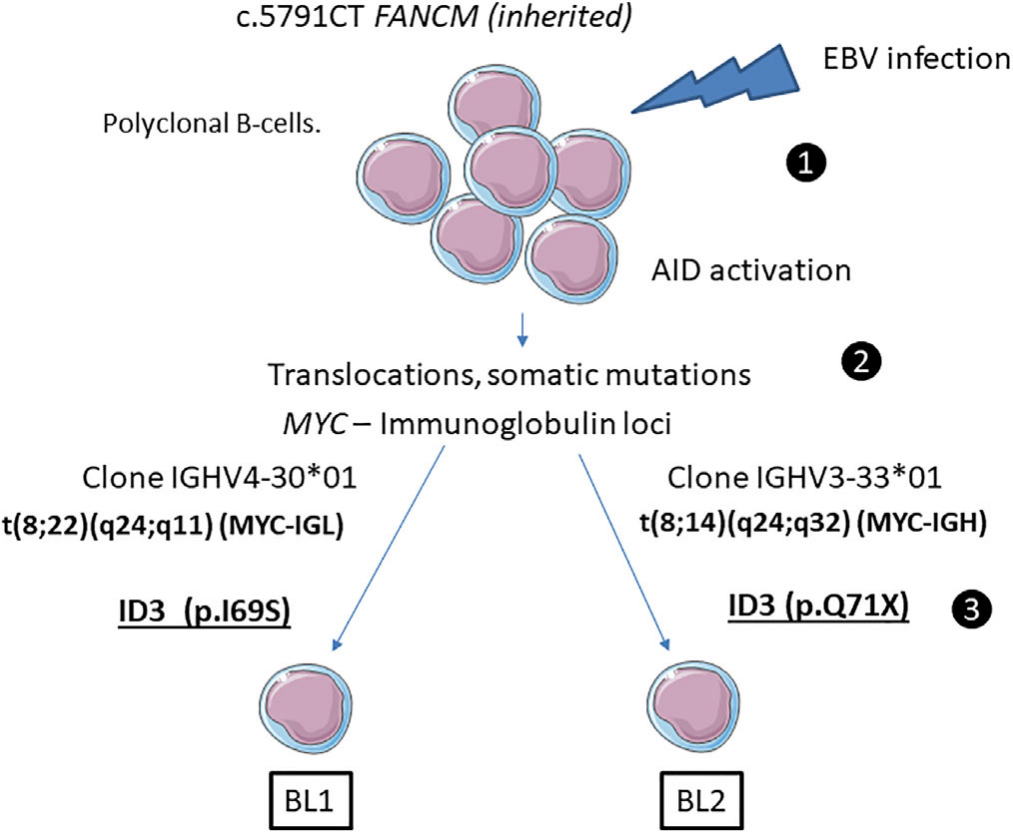
Schematic view of the different steps of the lymphomagenesis. 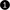 Polyclonal B‐cell infection by EBV. 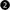 During EBV+ B‐cell maturation through germinal center transit, AID is expressed and favors *MYC/IG* loci (IGH or IGL) translocation and somatic mutations. 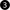 As *MYC* rearrangement and EBV infection, the *ID3* gene is targeted by a somatic mutational process shared by the two clonally recurrent Burkitt lymphoma (BL) and act synergistically with MYC [Color figure can be viewed at http://wileyonlinelibrary.com]

To verify the functional consequences of the c.5791C>T mutation, we performed reverse transcriptase‐polymerase chain reaction (RT‐PCR) with patient PBMC RNA. We confirmed that this mutation was also related to aberrant splicing (Supporting Information Figure S4) with the expression of the Δ22 allele. A qRT‐PCR assay indicated that the Δ22 and wild‐type alleles were equally expressed in the BL tumor cells (Supporting Information Figure S4). Of note, in an additional cohort of 29 BL cases with available tumor DNA, we did not find the *FANCM* c.5791C>T nonsense mutation (Supporting Information Table S3). However we cannot rule out other mutations targeting this gene. Biallelic inactivating mutations in *FANCM* favor early‐onset cancer, although the patients typically do not present congenital malformations or hematological disorder suggestive of FA.^25^ Importantly, our patient did not develop unusual toxicities during genotoxic treatments, including radiotherapy, suggesting the absence of an underlying FA condition. Consistently, primary fibroblast cells from the patients did not exhibit hypersensitivity to cross‐linking agent MMC, excluding the FA diagnosis. Overall the role of the heterozygous truncating *FANCM* mutations in the emergence of the two unrelated BL and MYC translocation is unclear. While we ruled out a bona fide Fanconi anemia, *FANCM* heterozygous variants have been associated to weak cancer predisposition by GWAS studies.^26^ Heterozygous loss of function mutations within the *FANCM* gene, including the c.5791C>T variant, were also significantly associated with familial breast cancer risk, with an overall odds ratio (OR) of 2.05.^27^ Despite a quantitative difference, the pattern of somatic mutations observed in BL1 and BL2 did not differ significantly, suggesting that BL1 and BL2 were sustained by a similar mutational process rather than arising as a consequence of a treatment side effect (Supporting Information Figure S5). To conclude, this unusual observation highlights the key events that lead to the emergence of genetically distinct BL types (Figure 1). The role of the inherited heterozygous truncating c.5791C>T *FANCM* mutation is uncertain and could be purely coincidental. *ID3* mutations are shared by the two clonally distinct diseases and represent a key secondary genetic event following *MYC* translocation. The *PI3KCD* mutation expands the spectrum of mutations targeting the PI3K pathway and offers potential therapeutic opportunities in BL.

## Supporting information

Supporting information
