# Supplementary material for: A recurrent clonally distinct Burkitt lymphoma case highlights genetic key events contributing to oncogenesis
Source: Genes Chromosomes Cancer. 2019 Mar 27;58(8):595–601. doi: 10.1002/gcc.22743 (PMC6790587; doi:10.1002/gcc.22743)
Supplement: Supplementary file 1 — Supporting information [file GCC-58-595-s001.docx]

**Supporting information**

**Penther et al.**

**A RECURRENT CLONALLY DISTINCT BURKITT LYMPHOMA CASE**

**HIGHLIGHTS GENETIC KEY EVENTS CONTRIBUTING TO ONCOGENESIS**

**1/ Supporting information Tables 1-6**

**Supporting information table 3**

**Supporting information Table 4.**

1. **PI3KC primers used of kinase domain sequencing**

| PIK3CD-ex16_17F | CTCGCTAGGTCCTGCTGG |
| --- | --- |
| PIK3CD-ex16_17R | TGGGAGGAACCCTTGTGG |
| PIK3CD-ex18_19F | CACAAGGGTTCCTCCCAC |
| PIK3CD-ex18_19R | CACAGACTCCCAGGCTGG |
| PIK3CD-ex20F | CTTTTTGGGGCACCATGAG |
| PIK3CD-ex20R | CGCTCCTTTGTTCTGACAGG |
| PIK3CD-ex21-22F | CAAGTCACAGGGCCAGATTC |
| PIK3CD-ex21-22R | GGAACCTCTGCCCTGTTCC |
| PIK3CD-ex23F | GGCTGGTTGGATGCAGAG |
| PIK3CD-ex23R | CTAGGTCACATTGCTCGGG |
| PIK3CD-ex24F | TTCAGTGACTCTGAAGTCCCC |
| PIK3CD-ex24R | CCGTTAGGTGCAGTTCAGG |

1. **Primers used to detect the c.5791C>T, p.Arg1931X FANCM variant and FANCM mRNA expression by TaqMan®**

**Supporting information table 5**

1. **Details of the somatic variants observed in BL1**

1. **Details of the somatic variants observed in BL2**

**Supporting information table 6**

1. **Details of the CNV observed in BL1**

| **Gene** | **Chr** | **Band** | **Type** | **Start** | **End** | **Gene description** |
| --- | --- | --- | --- | --- | --- | --- |
| OR4F29 | 1 | p36.33 | Gain | 367640 | 368634 | olfactory receptor, family 4, subfamily F, member 29 |
| PPIAL4C | 1 | q21.2 | Loss | 149553003 | 149553787 | peptidylprolyl isomerase A (cyclophilin A)-like 4C |
| ENAH | 1 | q42.12 | Gain | 225674537 | 225840844 | enabled homolog (Drosophila) |
| RYR2 | 1 | q43 | Gain | 237205505 | 237997288 | ryanodine receptor 2 (cardiac) |
| ZP4 | 1 | q43 | Gain | 238045705 | 238054094 | zona pellucida glycoprotein 4 |
| FAM90A26 | 4 | p16.1 | Gain | 9172135 | 9178453 | family with sequence similarity 90, member A26 |
| REXO1L11P | 8 | q21.2 | Gain | 86747543 | 86749570 | REX1, RNA exonuclease 1 homolog (S. cerevisiae)-like 11 |
| REXO1L10P | 8 | q21.2 | Gain | 86756718 | 86758745 | REX1, RNA exonuclease 1 homolog (S. cerevisiae)-like 10 |
| CNTNAP3 | 9 | p13.1 | Gain | 39072764 | 39288312 | contactin associated protein-like 3 |
| CNTNAP3 | 9 | p13.1 | Loss | 39072764 | 39288312 | contactin associated protein-like 3 |
| RABL6 | 9 | q34.3 | Gain | 139702374 | 139735639 | RAB, member RAS oncogene family-like 6 |
| C9orf172 | 9 | q34.3 | Gain | 139738867 | 139741797 | chromosome 9 open reading frame 172 |
| PHPT1 | 9 | q34.3 | Gain | 139743176 | 139745488 | phosphohistidine phosphatase 1 |
| MAMDC4 | 9 | q34.3 | Gain | 139745395 | 139755249 | MAM domain containing 4 |
| EDF1 | 9 | q34.3 | Gain | 139756571 | 139760738 | endothelial differentiation-related factor 1 |
| TRAF2 | 9 | q34.3 | Gain | 139776364 | 139821059 | TNF receptor-associated factor 2 |
| FBXW5 | 9 | q34.3 | Gain | 139834887 | 139839148 | F-box and WD repeat domain containing 5 |
| C8G | 9 | q34.3 | Gain | 139839698 | 139841426 | complement component 8, gamma polypeptide |
| LCN12 | 9 | q34.3 | Gain | 139844003 | 139849949 | lipocalin 12 |
| PTGDS | 9 | q34.3 | Gain | 139871956 | 139879887 | prostaglandin D2 synthase 21kDa (brain) |
| LCNL1 | 9 | q34.3 | Gain | 139876356 | 139880862 | lipocalin-like 1 |
| C9orf142 | 9 | q34.3 | Gain | 139886870 | 139888436 | chromosome 9 open reading frame 142 |
| CLIC3 | 9 | q34.3 | Gain | 139889087 | 139891255 | chloride intracellular channel 3 |
| ABCA2 | 9 | q34.3 | Gain | 139901686 | 139923367 | ATP-binding cassette, sub-family A (ABC1), member 2 |
| FUT7 | 9 | q34.3 | Gain | 139924626 | 139927462 | fucosyltransferase 7 (alpha (1,3) fucosyltransferase) |
| NPDC1 | 9 | q34.3 | Gain | 139933922 | 139940655 | neural proliferation, differentiation and control, 1 |
| ENTPD2 | 9 | q34.3 | Gain | 139942550 | 139948497 | ectonucleoside triphosphate diphosphohydrolase 2 |
| SAPCD2 | 9 | q34.3 | Gain | 139956581 | 139965040 | suppressor APC domain containing 2 |
| UAP1L1 | 9 | q34.3 | Gain | 139971953 | 139978991 | UDP-N-acteylglucosamine pyrophosphorylase 1-like 1 |
| MAN1B1 | 9 | q34.3 | Gain | 139981379 | 140003635 | mannosidase, alpha, class 1B, member 1 |
| DPP7 | 9 | q34.3 | Gain | 140004994 | 140009629 | dipeptidyl-peptidase 7 |
| GRIN1 | 9 | q34.3 | Gain | 140032842 | 140063207 | glutamate receptor, ionotropic, N-methyl D-aspartate 1 |
| LRRC26 | 9 | q34.3 | Gain | 140063210 | 140064503 | leucine rich repeat containing 26 |
| TMEM210 | 9 | q34.3 | Gain | 140065310 | 140066861 | transmembrane protein 210 |
| ANAPC2 | 9 | q34.3 | Gain | 140069236 | 140082989 | anaphase promoting complex subunit 2 |
| SSNA1 | 9 | q34.3 | Gain | 140083099 | 140084822 | Sjogren syndrome nuclear autoantigen 1 |
| TPRN | 9 | q34.3 | Gain | 140086069 | 140098645 | taperin |
| TMEM203 | 9 | q34.3 | Gain | 140098534 | 140100090 | transmembrane protein 203 |
| NDOR1 | 9 | q34.3 | Gain | 140100147 | 140111461 | NADPH dependent diflavin oxidoreductase 1 |
| RNF208 | 9 | q34.3 | Gain | 140114707 | 140116033 | ring finger protein 208 |
| C9orf169 | 9 | q34.3 | Gain | 140119087 | 140120763 | chromosome 9 open reading frame 169 |
| RNF224 | 9 | q34.3 | Gain | 140122018 | 140124090 | ring finger protein 224 |
| SLC34A3 | 9 | q34.3 | Gain | 140125209 | 140131006 | solute carrier family 34 |
| TUBB4B | 9 | q34.3 | Gain | 140135665 | 140138159 | tubulin, beta 4B class IVb |
| FAM166A | 9 | q34.3 | Gain | 140138036 | 140142222 | family with sequence similarity 166, member A |
| C9orf173 | 9 | q34.3 | Gain | 140145713 | 140147934 | chromosome 9 open reading frame 173 |
| NELFB | 9 | q34.3 | Gain | 140149625 | 140167998 | negative elongation factor complex member B |
| TOR4A | 9 | q34.3 | Gain | 140172201 | 140177093 | torsin family 4, member A |
| PTPN20A | 10 | q11.22 | Gain | 46549955 | 46641045 | protein tyrosine phosphatase, non-receptor type 20A |
| AGAP10 | 10 | q11.22 | Loss | 47191844 | 47239738 | ArfGAP with GTPase domain |
| NALCN | 13 | q33.1 | Gain | 101706130 | 102068843 | sodium leak channel, non-selective |
| PLD4 | 14 | q32.33 | Gain | 105391153 | 105399574 | phospholipase D family, member 4 |
| AHNAK2 | 14 | q32.33 | Gain | 105403581 | 105444694 | AHNAK nucleoprotein 2 |
| IGFLR1 | 19 | q13.12 | Gain | 36230058 | 36233354 | IGF-like family receptor 1 |
| U2AF1L4 | 19 | q13.12 | Gain | 36233365 | 36236346 | U2 small nuclear RNA auxiliary factor 1-like 4 |
| PSENEN | 19 | q13.12 | Gain | 36236015 | 36237911 | presenilin enhancer gamma secretase subunit |
| LIN37 | 19 | q13.12 | Gain | 36239262 | 36245420 | lin-37 homolog (C. elegans) |
| HSPB6 | 19 | q13.12 | Gain | 36245469 | 36248980 | heat shock protein, alpha-crystallin-related, B6 |
| C19orf55 | 19 | q13.12 | Gain | 36249044 | 36261930 | chromosome 19 open reading frame 55 |
| ZNF526 | 19 | q13.2 | Gain | 42724423 | 42732353 | zinc finger protein 526 |
| GSK3A | 19 | q13.2 | Gain | 42734338 | 42746777 | glycogen synthase kinase 3 alpha |
| ERF | 19 | q13.2 | Gain | 42751724 | 42759309 | Ets2 repressor factor |
| CIC | 19 | q13.2 | Gain | 42772689 | 42799949 | capicua transcriptional repressor |
| PAFAH1B3 | 19 | q13.2 | Gain | 42801185 | 42807698 | platelet-activating factor acetylhydrolase 1b |
| PRR19 | 19 | q13.2 | Gain | 42806250 | 42814973 | proline rich 19 |
| TMEM145 | 19 | q13.2 | Gain | 42817477 | 42829214 | transmembrane protein 145 |
| MEGF8 | 19 | q13.2 | Gain | 42829761 | 42882921 | multiple EGF-like-domains 8 |
| FAM182B | 20 | p11.1 | Loss | 25744102 | 25848861 | family with sequence similarity 182, member B |
| TPTE | 21 | p11.1 | Gain | 10906201 | 11029719 | transmembrane phosphatase with tensin homology |
| IGLV1-50 | 22 | q11.22 | Gain | 22681658 | 22682172 | immunoglobulin lambda variable 1-50 (non-functional) |
| IGLV9-49 | 22 | q11.22 | Gain | 22697539 | 22698084 | immunoglobulin lambda variable 9-49 |
| IGLV5-48 | 22 | q11.22 | Gain | 22707289 | 22707781 | immunoglobulin lambda variable 5-48 (non-functional) |
| IGLV1-47 | 22 | q11.22 | Gain | 22712087 | 22712608 | immunoglobulin lambda variable 1-47 |
| IGLV7-46 | 22 | q11.22 | Gain | 22723982 | 22724454 | immunoglobulin lambda variable 7-46 (gene/pseudogene) |
| IGLV5-45 | 22 | q11.22 | Gain | 22730355 | 22730874 | immunoglobulin lambda variable 5-45 |
| IGLV1-44 | 22 | q11.22 | Gain | 22735135 | 22735715 | immunoglobulin lambda variable 1-44 |
| IGLV7-43 | 22 | q11.22 | Gain | 22749356 | 22749827 | immunoglobulin lambda variable 7-43 |
| IGLV1-40 | 22 | q11.22 | Gain | 22764098 | 22764614 | immunoglobulin lambda variable 1-40 |
| IGLV5-37 | 22 | q11.22 | Loss | 22781876 | 22782371 | immunoglobulin lambda variable 5-37 |
| IGLV1-36 | 22 | q11.22 | Loss | 22786296 | 22786802 | immunoglobulin lambda variable 1-36 |
| ZNF280B | 22 | q11.22 | Loss | 22838767 | 22863505 | zinc finger protein 280B |
| ZNF280A | 22 | q11.22 | Loss | 22868060 | 22874613 | zinc finger protein 280A |
| PRAME | 22 | q11.22 | Loss | 22890123 | 22901768 | preferentially expressed antigen in melanoma |
| IGLV2-33 | 22 | q11.22 | Loss | 22930626 | 22931145 | immunoglobulin lambda variable 2-33 (non-functional) |
| IGLV3-32 | 22 | q11.22 | Loss | 22936998 | 22937501 | immunoglobulin lambda variable 3-32 (non-functional) |
| IGLV4-3 | 22 | q11.22 | Gain | 23213686 | 23214214 | immunoglobulin lambda variable 4-3 |
| IGLV3-1 | 22 | q11.22 | Gain | 23222886 | 23223576 | immunoglobulin lambda variable 3-1 |
| IGLL5 | 22 | q11.22 | Gain | 23229960 | 23238287 | immunoglobulin lambda-like polypeptide 5 |
| IGLC1 | 22 | q11.22 | Gain | 23237555 | 23238014 | immunoglobulin lambda constant 1 (Mcg marker) |
| IGLC2 | 22 | q11.22 | Gain | 23243156 | 23243617 | immunoglobulin lambda constant 2 (Kern-Oz- marker) |
| IGLC3 | 22 | q11.22 | Gain | 23248512 | 23248973 | immunoglobulin lambda constant 3 (Kern-Oz+ marker) |
| SRY | Y | p11.31 | Loss | 2654896 | 2655740 | sex determining region Y |
| RPS4Y1 | Y | p11.31 | Loss | 2709527 | 2800041 | ribosomal protein S4, Y-linked 1 |
| ZFY | Y | p11.31 | Loss | 2803112 | 2850547 | zinc finger protein, Y-linked |
| TGIF2LY | Y | p11.2 | Loss | 3447082 | 3448082 | TGFB-induced factor homeobox 2-like, Y-linked |
| PCDH11Y | Y | p11.2 | Loss | 4868267 | 5610265 | protocadherin 11 Y-linked |
| TSPY2 | Y | p11.2 | Loss | 6114264 | 6117060 | testis specific protein, Y-linked 2 |
| AMELY | Y | p11.2 | Loss | 6733959 | 6742068 | amelogenin, Y-linked |
| TBL1Y | Y | p11.2 | Loss | 6778727 | 6959724 | transducin (beta)-like 1, Y-linked |
| TSPY4 | Y | p11.2 | Loss | 9175073 | 9177893 | testis specific protein, Y-linked 4 |
| TSPY8 | Y | p11.2 | Loss | 9195406 | 9218479 | testis specific protein, Y-linked 8 |
| TSPY3 | Y | p11.2 | Loss | 9236030 | 9307357 | testis specific protein, Y-linked 3 |
| TSPY1 | Y | p11.2 | Loss | 9236076 | 9307357 | testis specific protein, Y-linked 1 |
| TSPY6P | Y | p11.2 | Loss | 9324922 | 9327689 | testis specific protein, Y-linked 6, pseudogene |
| TSPY10 | Y | p11.2 | Loss | 9365489 | 9368291 | testis specific protein, Y-linked 10 |
| FAM197Y1 | Y | p11.2 | Loss | 9374241 | 9384693 | family with sequence similarity 197, Y-linked, member 1 |
| SLC9B1P1 | Y | q11.21 | Loss | 13496241 | 13524717 | solute carrier family 9, subfamily B |
| USP9Y | Y | q11.21 | Loss | 14813160 | 14972764 | ubiquitin specific peptidase 9, Y-linked |
| DDX3Y | Y | q11.21 | Loss | 15016019 | 15032390 | DEAD (Asp-Glu-Ala-Asp) box helicase 3, Y-linked |
| UTY | Y | q11.221 | Loss | 15360259 | 15592553 | ubiquitously transcribed tetratricopeptide repeat |
| TMSB4Y | Y | q11.221 | Loss | 15815447 | 15817904 | thymosin beta 4, Y-linked |
| VCY | Y | q11.221 | Loss | 16097652 | 16098393 | variable charge, Y-linked |
| VCY1B | Y | q11.221 | Loss | 16168097 | 16168838 | variable charge, Y-linked 1B |
| NLGN4Y | Y | q11.221 | Loss | 16634518 | 16957530 | neuroligin 4, Y-linked |
| CDY2B | Y | q11.222 | Loss | 19989290 | 19992100 | chromodomain protein, Y-linked, 2B |
| CDY2A | Y | q11.222 | Loss | 20137667 | 20140477 | chromodomain protein, Y-linked, 2A |
| HSFY1 | Y | q11.222 | Loss | 20708557 | 20750849 | heat shock transcription factor, Y-linked 1 |
| HSFY2 | Y | q11.222 | Loss | 20893326 | 20990548 | heat shock transcription factor, Y linked 2 |
| KDM5D | Y | q11.222 | Loss | 21865751 | 21906825 | lysine (K)-specific demethylase 5D |
| EIF1AY | Y | q11.223 | Loss | 22737611 | 22755040 | eukaryotic translation initiation factor 1A, Y-linked |
| RPS4Y2 | Y | q11.223 | Loss | 22918050 | 22942918 | ribosomal protein S4, Y-linked 2 |
| RBMY1B | Y | q11.223 | Loss | 23673224 | 23687672 | RNA binding motif protein, Y-linked, family 1, member B |
| RBMY1A1 | Y | q11.223 | Loss | 23673258 | 23711212 | RNA binding motif protein, Y-linked, family 1, member A1 |
| RBMY1E | Y | q11.223 | Loss | 24026223 | 24064214 | RNA binding motif protein, Y-linked, family 1, member E |
| RBMY1D | Y | q11.223 | Loss | 24026223 | 24040673 | RNA binding motif protein, Y-linked, family 1, member D |
| RBMY1F | Y | q11.223 | Loss | 24314689 | 24329129 | RNA binding motif protein, Y-linked, family 1, member F |
| RBMY1J | Y | q11.223 | Loss | 24454970 | 24564028 | RNA binding motif protein, Y-linked, family 1, member J |
| DAZ1 | Y | q11.223 | Loss | 25275502 | 25345241 | deleted in azoospermia 1 |
| DAZ2 | Y | q11.223 | Loss | 25365594 | 25437503 | deleted in azoospermia 2 |
| CDY1B | Y | q11.223 | Loss | 26191376 | 26194166 | chromodomain protein, Y-linked, 1B |
| DAZ3 | Y | q11.23 | Loss | 26909216 | 26959626 | deleted in azoospermia 3 |
| DAZ4 | Y | q11.23 | Loss | 26980008 | 27053183 | deleted in azoospermia 4 |
| CDY1 | Y | q11.23 | Loss | 27768264 | 27771049 | chromodomain protein, Y-linked, 1 |

1. **Details of the CNV observed in BL2**

| **Gene** | **Chr** | **Band** | **Type** | **Start** | **End** | **Gene description** |
| --- | --- | --- | --- | --- | --- | --- |
| GPR89B | 1 | q21.2 | Loss | 147400506 | 147465753 | G protein-coupled receptor 89B |
| PPIAL4C | 1 | q21.2 | Loss | 149553003 | 149553787 | peptidylprolyl isomerase A (cyclophilin A)-like 4C |
| ANXA8L1 | 10 | q11.22 | Gain | 47157983 | 47174093 | annexin A8-like 1 |
| FAM25B | 10 | q11.22 | Gain | 47177204 | 47181681 | family with sequence similarity 25, member B |
| AGAP10 | 10 | q11.22 | Loss | 47191844 | 47239738 | ArfGAP with GTPase domain |
| NA | 22 | q11.21 | Gain | 18721427 | 18745407 | Uncharacterized protein |
| RIMBP3C | 22 | q11.21 | Gain | 21899646 | 21905750 | RIMS binding protein 3C |

**2/ Supporting information figures 1-8**
